# The influence of biased technical progress on employment scale of the circulation industry: Evidence from China

**DOI:** 10.1371/journal.pone.0300126

**Published:** 2024-03-26

**Authors:** Zhenqiu Wu, Biqing Yue, Yue Dai, Yujin Ge, Guangmei Lu, Fei Zu

**Affiliations:** School of Business Administration, Zhongnan University of Economics and Law, Wuhan City, Hubei Province, China; Wuhan Institute of Technology, CHINA

## Abstract

As a leading industry in the national economy, circulation industry can not only guide production and consumption, but also play a vital role in absorbing employment. With the progress of science and technology, technical change has penetrated into the circulation industry of China, which has not only improved its development, but also affected its employment. This paper uses the standardized supply-side system approach to measure the biased technical progress of circulation industry in China and investigates the influence of the biased technical progress index on the employment scale of circulation industry in China with panel regression model. We find that the overall technical progress in China’s circulation industry during 2004–2018 is biased toward capital, and the elasticity of substitution between capital and labor is less than 1. We also find capital-biased technical progress in China’s circulation industry is negatively related to the overall employment scale of circulation industry. The heterogeneity analysis indicates that the employment of non-state-owned units in circulation industry is significantly negatively affected by capital-biased technical progress, while state-owned units doesn’t.

## 1. Introduction

China, as a large developing country, has made great achievements in the sustained and rapid development of its national economy since implementation of reform and opening up policies. It has always been the goal of China’s macroeconomic policy to solve the problem of unemployment to the greatest extent, as a top priority for the people’s livelihood in developing countries. However, with the relentless march of technical progress, employment problems have become increasingly prominent in China. Every year, about 25–35 million higher and secondary education graduates, surplus labor force transferred from rural areas, and urban unemployed people need employment, while about 13–16 million urban jobs are provided each year. According to The Statistical Communique on the Development of Human Resources and Social Security in 2019, 774.71 million people were employed at the end of 2019, 13.52 million new urban employment opportunities were generated, and 9.45 million urban registered unemployed people were registered at the end of 2019. The registered urban unemployment rate was 3.6 percent, and the surveyed urban unemployment rate was 5.2 percent nationwide. In 2020, due to the severe impact of COVID-19, the employment situation was even worse. In 2020, number of new urban jobs was 11.86 million, 1.66 million fewer than the previous year.

As the main topic of economics, employment has been paid close attention by economists. Neoclassical economics believes that the labor market will achieve automatic equilibrium in the long run, and there will be no unemployment. However, the long-term and large-scale unemployment caused by the great depression in 1929–1933 broke this view. Then, Keynes denied the Neoclassical school’s assumption of full employment and emphasized the importance of state intervention. In 1958, Phillips discovered an alternating relationship between unemployment rate and growth rate of monetary wages, based on empirical statistics from England spanning 1861 to 1913. In 1962, American economist Okun proposed the well-known “Okun’s law” by studying American economic data. This Law approximately describes the correlation between unemployment rate and potential GDP, indicating that for every 1% increase in unemployment above the natural rate, there will be a corresponding decrease of 2% in real GDP relative to potential. Based on Keynesianism, New-Keynesian economists affirmed the importance of government intervention in labor market. The New-Classical Macroeconomics believes that Keynesian economic policies are harmful, including those that intervene in the labor market. New-Classical macroeconomists point out that there is no Phillips curve in the short term. The views of various economic schools on the labor market and employment have certain reference significance for solving the employment problem.

Some economists believe that the service industry has the function of an “employment machine”. Recently, the prominent employment problem in China has highlighted the importance of the service industry. As the fundamental and leading industry of national economy, the circulation industry (including wholesale and retail industry; accommodation and catering industry; transportation, storage and postal industry) is an important pillar industry in the service industry, which plays a more vital role in absorbing employment. The full-year 2019 of the third industry employment accounted for 47.4%, and the circulation industry absorbed employment of 216.463 million people, about 59.0% of the third industry employment, which shows the contribution of circulation industry in absorbing employment. Therefore, when further expanding the employment capacity and realizing full employment, we must not ignore the important role of circulation industry. However, with the development of the economy and the progress of science and technology, China’s circulation industry informatization level, equipment, facilities and personnel quality continues to improve. It naturally brings about a profound question: on the one hand, all kinds of technical progress increase the volume of circulation business and reduces the cost of starting a business, which will attract more employees and entrepreneurs. On the other hand, the biased technical progress that dominates the circulation industry may “crowd out” employment. The so-called biased technical progress is relative to neutral technical progress. The theory of neutral technical progress believes that technical progress affects the production efficiency and input ratio of all factors in the same proportion, while the theory of biased technical progress believes that when the marginal output growth rate of one factor is higher than other factors, technical progress is biased to this factor [1]. Then, the overall impact of the biased technical progress in China’s circulation industry on employment is an urgent need for a comprehensive and in-depth research. From the perspective of biased technical progress, it is of great theoretical and practical significance to study the influence of technical progress on circulation industry’s employment scale and promote theoretical study on the correlation between biased technical progress and employment, to deeply reveal the mechanism of biased technical progress on circulation industry’s employment scale and to provide a reference for the labor department to expand the employment capacity in the circulation industry for decision making.

## 2. Literature review

### 2.1 Studies of biased technical progress

Technical progress can bring about the improvement of factor allocation efficiency and production efficiency [2], but whether its impact on different factors is homogeneous or not, there are different views in different periods. In the 1930s and 1940s, most scholars believed that technical progress was neutral, and the theory of neutral technical progress was popularized during this period. At about the same time, Hicks [3] proposed the idea of biased technical progress, but was drowned out. Solow [4] and Samuelson [5] both used data to verify the existence of neutral technical progress, and thus, this view of unbiased technical progress was widely accepted. However, with the development of science and technology, capital such as machinery and equipment is no longer just the embodiment of capital, but contains the factors of technical progress, becoming more automated, informationized and intelligent, that is, technical progress is no longer neutral, but has an asymmetric impact on the marginal output of capital and labor, resulting in the bias of technical progress [6]. At the same time, with the deepening of the research on technical progress, economists have gradually found that technical progress is not neutral. [7] discussed the bias of technical progress, but the academic circle did not recognize it due to the lack of a certain micro basis. In the 1990s, the academic circles paid more attention to the research on biased technical progress until Acemoglu [1] first started from the perspective of pursuing profit maximization. Furthermore, he integrated Hicks [8] and other thoughts on biased technical progress into the mathematical economic model based on his endogenous economic growth model.

This paper finds the two effects of biased technical progress, price effect and market scale effect. That is, when the substitution elasticity between the two input factors is less than 1, the price effect plays a more important role, which is shown as the factor that technical progress tends to be relatively scarce. When the substitution elasticity of the two input factors is greater than 1, the market scale effect plays a more important role, which shows that the technical progress tends to the relatively abundant elements, thus finding the micro basis for the biased technical progress. Since then, the academic circle has initiated a research upsurge on biased technical progress, and the theory of biased technical progress has been gradually enriched and improved. Scholars use the theory of biased technical progress to explain a series of unbalanced economic problems such as labor income gap and income distribution [9–12]. In recent years, scholars have applied biased technical progress widely, such as combining it with total factor productivity [13–19], economic growth [20,21], industrial structure upgrading and change [22], employment and unemployment [23,24], high-quality economic development [25]. Some scholars have also included energy into the input factor and explored the technology bias among the factor groups [26,27] and the bias of green technology progress [28].

### 2.2 Studies on the impact of biased technical progress on employment

Regarding the influence of biased technical progress on the scale of employment, the existing literature mainly includes: Acemoglu [29] used theoretical analysis to prove that the increase of wages could promote the rise of labor income share. However, the characteristic that technical progress was biased towards capital would restrain the rise of labor income share to some extent, thus bring about the reduction of employment scale. Abbott, Tarpb, and Wu [30] found that structural transformation is part of the reason for employment growth, while the characteristics of technical progress and capital bias are the reasons for the large gap in employment growth. Miao and Wan [31] thought that the capital-biased technology progress in China made the surplus labor force produced in the process of economic transition not effectively be utilized, which led to a large number of unemployed people in China. Wu [32] found that biased technical progress inhibited the growth of employment and further aggravated the differentiation of China’s labor market. Duan and Zhong [33] used the data from 1978 to 2013 to measure China’s biased technical progress index. They found that capital-biased technical progress is the major reason why the employment growth rate has lagged behind economic growth. Wu et al. [34] adopted the data of Chinese manufacturing enterprises from 2007 to 2018 to explore the influence of capital-biased technical progress on manufacturing employment growth and found that the growth rate of capital-labor ratio was negatively correlated with the growth rate of manufacturing employment. Pi [35] believed that capital-biased technical progress would gradually replace or squeeze out the low productivity sectors with high productivity, thus reducing the overall employment scale. Wang [36] used the industry data from 2005–2013 to study the direction of technical progress of third industry sub-sectors and its influence on employment by using the transcendental logarithmic production function. He also found that the technical progress direction of real estate, finance, and wholesale and retail industry in China was capital-biased technology progress, and capital-biased technology progress reduced the employment volume of these industries. The accommodation and catering industry and other industries are characterized by labor-biased technical progress, which promotes the employment of these industries. Zhao and Dong [37] paid attention to the influence of materialized technology progress on different skilled labor employment. It was found that from 2002 to 2015, the materialized technology progress inhibited the overall employment growth in China. However, it had different effects on different levels of skilled labor employment, which showed strong inhibition of low-skilled labor employment and played a role in promoting high-skilled labor employment.

From the above analysis, it can be seen that domestic and foreign scholars’ studies on the influence of biased technical progress on employment are relatively mature at the macro and meso levels, mainly focusing on the study of the employment scale. In addition, the subject of existing literature research is mainly focused on the national level or the level of the manufacturing industry, with few specific studies on the third industry and even fewer studies on the sub-sectors of the third industry. The innovations of this paper are as follows. First, this paper adopts provincial industry-level data to measure the bias of technical progress in China’s circulation industry and sub-sectors with the standardized supply-side system approach, clarifies the types of capital-labor relationship in China’s circulation industry and sub-sectors, and conducts a quantitative study on the influence of biased technical progress index on employment size in China’s circulation industry to fill the gaps left by the existing studies. Second, from the perspective of heterogeneity, we conduct panel regression analysis on the effects of biased technical progress on the employment scale of different ownership, different education levels, urban and rural areas, different sub-sectors, and different regions in the circulation industry, expanded the scope of the study. It provides empirical support for the national decision-making departments to solve the employment problem of circulation industry from the dimension of biased technical progress and relieve the employment pressure to stimulate further coordinated development between biased technical progress and employment of circulation industry.

## 3. Analysis of the mechanism of biased technical progress on employment in circulation industry

As for the production function of the two input factors of capital and labor, biased technical progress can only be divided into capital-biased technical progress and labor-biased technical progress. So, the following separately analyzes the mechanism of capital-biased technical progress and labor-biased technical progress on circulation industry employment.

### 3.1 Mechanism of capital-biased technical progress on circulation industry employment

According to research by Dai and Xu [38], capital-biased technical progress can be divided into two situations. The first is that the elasticity of substitution between capital and labor is greater than 1 (the relationship between capital and labor is substitution relationship), and technical progress is capital-enhanced. The second is that the elasticity of substitution between capital and labor is less than 1 (the relationship between capital and labor is complementary), and technical progress is labor-enhanced. The first situation: capital-enhanced technical progress means that the expansion of capital input due to the technical progress, that is, owing to technical progress, 1 unit of capital input will bring more than one time of capital at work, and the capital efficiency parameter expresses the multiple of the capital expansion. When the capital efficiency parameter is greater than the labor efficiency parameter, and capital and labor are a substitute relationship, the circulation enterprises will inevitably increase capital input and reduce labor input, it will have a “destructive effect” on employment in the circulation industry. The second situation: labor-enhanced technical progress means that the expansion of labor input due to technical progress, that is, owing to technical progress, 1 unit of labor input will bring more than one time of labor at work, and the labor efficiency parameter expresses the multiple of labor expansion. When the capital efficiency parameter is smaller than the labor efficiency parameter, and capital and labor are in complementary relationship, if consumers’ demand for the circulation industry is fixed or decreases, resulting in a fixed or decreased output of the circulation industry, the improvement of the labor efficiency parameter of circulation industry will make enterprises reduce labor input, which will have a “destructive effect” on employment. If consumers’ demand for the circulation industry rises, leads to an increase in the output of circulation industry, it may increase labor input, which will have a “creative effect” on employment in the circulation industry. However, if the output of circulation industry does not rise sufficiently (the increase in output is not larger than the increase in labor efficiency), it is possible that the enterprise will not increase labor input, and this will produce a “destructive effect” of employment in circulation industry. Of course, because capital and labor are complementary, when enterprises increase or decrease labor, they will inevitably increase or decrease capital investment accordingly. In other words, there may be both a “destructive effect” and a “creative effect” of employment in the second situation.

### 3.2 Mechanism of labor-biased technical progress on circulation industry employment

**Fig 1:**
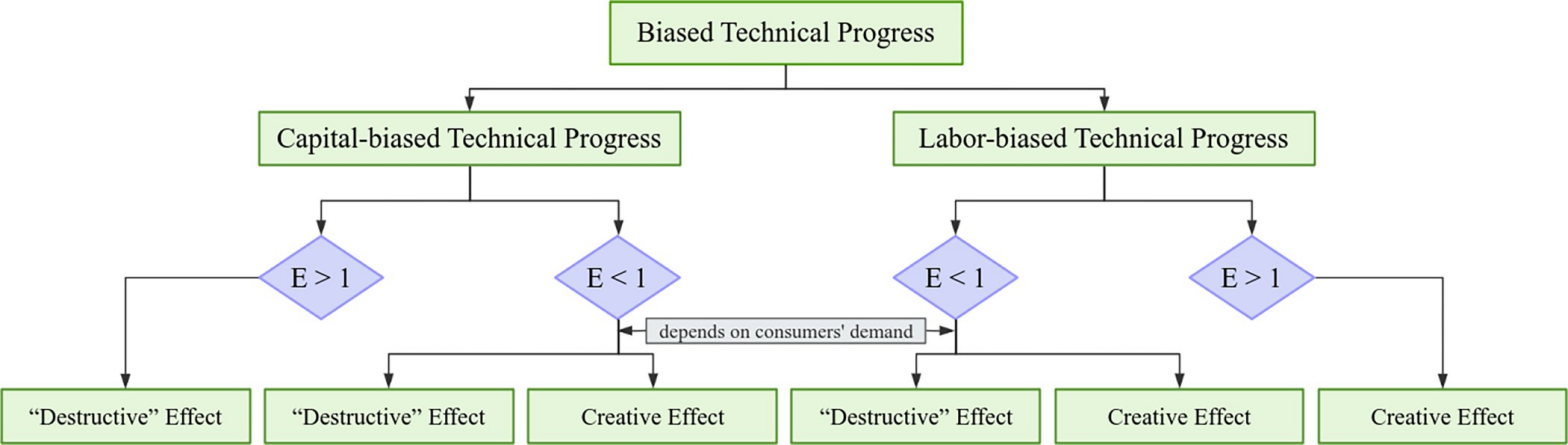
Effect mechanism of the biased technical progress on employment. Notes: E means the elasticity of substitution between capital and labor.

Labor-biased technical progress also can be divided into two situations. The first is that the elasticity of substitution between capital and labor is greater than 1 (the relationship between capital and labor is a substitution relationship), and technical progress is labor-enhanced. The second is that the elasticity of substitution between capital and labor is less than 1 (the relationship between capital and labor is complementary), and technical progress is capital-enhancing. The first situation: labor-enhanced technical progress, when the labor efficiency parameter is greater than capital efficiency parameter, and capital and labor are substitute relationship, the circulation enterprises will inevitably increase labor input, which will have a “creative effect” on employment in the circulation industry. The second situation: capital-enhanced technical progress, when the capital efficiency parameter is greater than labor efficiency parameter, and the relationship between capital and labor is complementary, if consumers’ demand for circulation industry is fixed or decreases, resulting in a fixed or decreased output of circulation industry, the improvement of capital efficiency parameters in circulation industry will make enterprises reduce capital input. since capital and labor are complementary, the enterprise will also reduce labor input at the same time, which will have a “destructive effect” on employment. If consumers’ demand for the circulation industry rises, leads to an increase in the output of circulation industry, it may increase capital input and labor input simultaneously, which will have a “creative effect” on employment. However, if the output of the circulation industry does not rise sufficiently (output rises smaller than capital efficiency rises), it is possible that the enterprise will not increase capital input and will not increase labor input simultaneously, which will produce a “destructive effect” of employment in the circulation industry. In other words, there may be both a “destructive effect” and a “creative effect” of employment in the second situation. The mechanism of the biased technical progress on employment discussed above can be abstracted into the Fig 1.

## 4. Measurement and analysis of the biased technical progress index of circulation industry

### 4.1 Measurement method of biased technical progress index

Analyzing previous scholars’ research [1,39–41], we found that the factor-enhanced CES production function is used more to measure biased technical progress. The reason is that the relative marginal output change of factors in the definition of biased technical progress is not only related to the factor efficiency, but also related to the substitution elasticity between factors. The factor-enhanced CES production function contains both the factor substitution elasticity index and the factor-enhanced technical progress parameters, which can reflect the direction of technical progress. At the same time, the estimation results of this method are robust and accurate. Therefore, the factor-enhanced CES production function is an important model for the study of biased technical progress.

The factor-enhanced CES production function is:

$$Y_{t}={\left[(1‐\alpha ){\left(A_{t}L_{t}\right)}^{\frac{\sigma ‐1}{\sigma }}+\alpha {\left(B_{t}K_{t}\right)}^{\frac{\sigma ‐1}{\sigma }}\right]}^{\frac{\sigma }{\sigma ‐1}}$$ (1)

Where Y_t_ is the actual output level of circulation industry, K_t_ and L_t_ represent the capital and labor input of the circulation industry, respectively. A_t_ and B_t_ represent the circulation industry labor efficiency and capital efficiency, respectively. α∈(0,1) is the capital intensity of the circulation industry, and σ∈(0,+∞) is the substitution elasticity of labor and capital in circulation industry. The marginal products of labor and capital are:

$${MP}_{L}=\frac{\partial Y}{\partial L}=(1‐\alpha ){\left(\frac{Y_{t}}{L_{t}}\right)}^{\frac{1}{\sigma }}{\left(A_{t}\right)}^{\frac{\sigma ‐1}{\sigma }}$$ (2)

$${MP}_{K}=\frac{\partial Y}{\partial K}=\alpha {\left(\frac{Y_{t}}{K_{t}}\right)}^{\frac{1}{\sigma }}{\left(B_{t}\right)}^{\frac{\sigma ‐1}{\sigma }}$$ (3)

According to Formulas (2) and (3), the ratio of marginal output of capital to labor can be obtained as:

$$\Delta_{t}=\frac{\partial Y/\partial K}{\partial Y/\partial L}=\frac{\alpha }{1‐\alpha }{\left(\frac{B_{t}}{A_{t}}\right)}^{\frac{\sigma }{\sigma ‐1}}{\left(\frac{L_{t}}{K_{t}}\right)}^{\frac{1}{\sigma }}$$ (4)

The meaning of Formula (4) is: when the proportion of capital and labor input is constant, if technical progress causes Δ_*t*_ to rise, then technical progress is manifested as capital-biased technical progress; if technical progress causes Δ_*t*_ to decrease, then technical progress is manifested as labor-biased technical progress; if technical progress does not affect Δ_*t*_, technical progress appears as neutral technical progress.

The above method provides a quantitative method for judging the direction of biased technical progress—the index of biased technical progress (D_t_) [38].


$$D_{t}=\frac{1}{\Delta }\frac{\partial \Delta }{\partial (B/A)}\frac{d(B/A)}{\mathrm{dt}}=\frac{\sigma ‐1}{\sigma }(\frac{A}{B})\frac{d(B_{t}/A_{t})}{\mathrm{dt}}$$ (5)

The economic meaning of D_t_ is the rate of change of the marginal output ratio of capital and labor resulting from technical progress.

At present, there are three main estimation methods for elasticity of substitution, inference method, single equation model estimation method and normalized supply-side system approach. Among them, the inference method cannot estimate the specific value of the elasticity of substitution; The estimation equations adopting the single equation model estimation method include CES production function, first order condition equation of capital and labor demand for maximizing enterprise profits and translog cost function. Any of these three equations requires strict restrictions on technical progress. Since the input of production factors is influenced by the relative price of factors, the estimation of any single equation exists systematic deviation [1,42]. Normalized supply-side system approach takes the standardized CES production function and first order conditions of capital and labor demand as a system to estimate. Because the elasticity of substitution and the parameter of the rate of technical progress in these three equations affect each other, so that the identifiable problem of structural parameter is solved, then the robust estimation of substitution elasticity and the index of biased technical progress can be obtained.

This paper adopts normalized supply-side system approach to insert the representation related to factor efficiency into the production function, find the first order conditions for maximizing profits and obtain the nonlinear simultaneous equations consisting of production function (6), labor demand function (7) and capital demand function (8) under optimal conditions:

$$\ln\left(\frac{Y_{t}}{\bar{Y}}\right)=\ln\left(\xi \right)+\frac{\sigma }{\sigma ‐1}\ln\left\{\begin{array}{c} (1‐\alpha ){\left\{\frac{L_{t}}{\bar{L}}\exp[\bar{t}\frac{\gamma_{L}}{\lambda_{L}}({\left(\frac{t}{\bar{t}}\right)}^{\lambda_{L}}‐1)]\right\}}^{\frac{\sigma ‐1}{\sigma }}\\ +\alpha {\left\{\frac{K_{t}}{\bar{K}}\exp[\bar{t}\frac{\gamma_{K}}{\lambda_{K}}({\left(\frac{t}{\bar{t}}\right)}^{\lambda_{K}}‐1)]\right\}}^{\frac{\sigma ‐1}{\sigma }} \end{array}\right\}$$ (6)

$$\ln\left(\frac{w_{t}L_{t}}{Y_{t}}\right)=\ln(1‐\alpha )+\frac{\sigma ‐1}{\sigma }\ln\left(\xi \right)‐\frac{\sigma ‐1}{\sigma }\ln(\frac{Y_{t}/\bar{Y}}{L_{t}/\bar{L}})+\frac{\sigma ‐1}{\sigma }\bar{t}\frac{\gamma_{L}}{\lambda_{L}}\left({\left(\frac{t}{\bar{t}}\right)}^{\lambda_{L}}‐1\right)$$ (7)

$$\ln\left(\frac{r_{t}K_{t}}{Y_{t}}\right)=\ln\;\alpha +\frac{\sigma ‐1}{\sigma }\ln\left(\xi \right)‐\frac{\sigma ‐1}{\sigma }\ln(\frac{Y_{t}/\bar{Y}}{K_{t}/\bar{K}})+\frac{\sigma ‐1}{\sigma }\bar{t}\frac{\gamma_{K}}{\lambda_{K}}\left({\left(\frac{t}{\bar{t}}\right)}^{\lambda_{K}}‐1\right)$$ (8)

Where w_L_ and r_L_ are labor price and capital price, $\bar{Y},\bar{K},\bar{L},\bar{t}$ are the mean value of Y, K, L, t. γ_L_ and γ_K_ are technical growth parameters, and λ_L_ and λ_K_ are technical curvatures. Since the CES production function is nonlinear, [42] introduced a scale factor ξ to solve the problem of the uncertain relationship between the initial value of output level and the initial value of factor input, and make $Y_{0}=\xi \bar{Y}$, $K_{0}=\bar{K}$, $L_{0}=\bar{L}$.

We make use of regression equation which is made up of Eqs (6), (7) and (8) to obtain the values of each parameter, substitute data such as elasticity of substitution, circulation industry output, labor and capital input into Eq (5), and obtain circulation industry and subdivision’s biased technical progress index.

### 4.2 Data source and indicator description

As China has repeatedly adjusted the division of industry sectors, the “Classification of National Economic Industries” (GB/T4754-2002) promulgated in 2003 has made the industry classifications before and after 2003 be quite different, and each province uses the new industry classification for statistics. There is a certain time lag, so this paper adopts the panel data of 30 provinces, municipalities, and autonomous regions in the circulation industry from 2004 to 2018 as research object. Tibet is deleted because there is much data missing. In this paper, we select data from 30 provinces, municipalities, and autonomous regions in China. Data from Tibet are discarded because of the serious missing data, which is not conducive to the subsequent analysis. In addition, data from Hong Kong, Macao, and Taiwan among 34 provincial administrative units in China are discarded. This paper selects the wholesale and retail industry, accommodation and catering industry, transportation, storage and postal industry, the three sub-sectors of the circulation industry to represent China’s circulation industry, and use the standardized supply-side three equations to measure the bias of technical progress of China’s circulation industry. It mainly involves five variables: circulation industry output Y, labor input L, capital input K, labor income share 1-S, and capital income share S.

First, the output of circulation industry (Y). This paper selects the total value-added of the wholesale and retail industry, accommodation and catering industry, transportation, storage and postal industry from 2004 to 2018 to represent the output of circulation industry. For the purpose of eliminating the impact of price factors, this paper uses the third industry value-added deflator of each province to adjust, and obtains the actual value-added of circulation industry and each sub-sector at a constant price in 2004. Among them, the data of the value-added of circulation industry in 2018 in some regions is missing, and this paper uses the regression method to fill it up.

Second, labor input (L). That is, employees of circulation industry, specifically. We use the sum of the year-end employment of urban collective units, urban state-owned units, other urban units, urban private units and individuals, and rural private units and individuals in circulation industry to represent the labor input in circulation industry.

Third, capital investment (K). That is, the capital stock of circulation industry, which is measured by perpetual inventory method, specifically: $K_{t}=I_{t}+(\mathrm{1-\delta})K_{\mathrm{t-1}}$, where K_t_ is the current capital stock of the circulation industry, K_t-1_ is the previous capital stock of circulation industry, and I_t_ is the current investment amount of the circulation industry, δ is the depreciation rate. Taking into account the influence of inflation, this paper adopts each province’s fixed asset price index to convert to get actual investment amount at constant prices in 2004. The capital stock in the base year of 2004 uses the steady-state method proposed by Harberger [43], and its estimation formula is:

$$K_{\mathrm{i,t-1}}=I_{\mathrm{i,t}}/\left(g_{\mathrm{i,t}}+\delta_{\mathrm{i,t}}\right)$$ (9)

The choice of capital depreciation rate δ: the three sub-sectors of the circulation industry have large differences in the use of fixed-assets, and the degree and cycle of depreciation abrasion are also different. According to the summary of previous literature, this paper adopts the industry-recognized capital depreciation rate of 4% in service industry [44]. Considering the influence of business cycle fluctuations, the average growth rate of output over a period recommended by Harberger [43] is expressed by g_i,t_. This paper adopts the average growth rate of actual value-added of circulation industry in each province from 2004 to 2018 to measure it.

Fourth, labor income share (1-S_i,t_) and capital income share (S_i,t_). When calculating the labor reward in the circulation industry, this paper first calculates the labor reward in each sub-sector and then sums up the data in each sub-sector to obtain the labor reward in the circulation industry. The method of calculating labor reward in each sub-sector is as follows:

$${LC}_{it}={uwage}_{it}\times {uemp}_{it}+{pwage}_{it}\times {pemp}_{it}$$ (10)

Among them, *uwage*_*it*_、*uemp*_*it*_、*pwage*_*it*_ and *pemp*_*it*_ represent the average wage of employees in urban units, the number of employees in urban units, the average wage of employees in urban private units and the number of employees in private units and individuals in each sub-sector respectively.

Since the average salary of employees in urban private units in each sub-sector only has data from 2009 to 2018, we calculate the data from 2004 to 2008 according to the comprehensive ratio from 2009 to 2018. The specific algorithm is as follows:

$${pwage}_{it^{\prime }}={uwage}_{it^{\prime }}\times \left(\sum_{t=2009}^{2018}{pwage}_{it}/\sum_{t=2009}^{2018}{uwage}_{it}\right),\;t\prime \in (2004,2008)$$ (11)

The labor share and capital share of the circulation industry are calculated as follows:

$$1-S_{it}=\sum {LC}_{it}/\sum {VA}_{it}$$ (12)

$$S_{it}=1-\sum {LC}_{it}/\sum {VA}_{it}$$ (13)

Among them, the labor reward of each sub-sector is deflated using the consumer price index to obtain the actual labor reward of each sub-sector with 2004 as base year, and the added value (*VA*_*it*_) is calculated by the value-added index of tertiary industry to get the actual added value based on 2004.

### 4.3 Analysis of biased technical progress in circulation industry

First, we adopt feasible generalized nonlinear least squares method (FGNLS) and estimate the substitution elasticity of circulation industry as a whole and sub-sector. Since the nonlinear estimation is very sensitive to the initial values of parameters, so we adopt “global optimization” method recommended by Klump et al. [42]. ξ is theoretically close to 1, so set its initial value ξ = 1. The value of αis theoretically equal to mean value of capital income share, so its initial value is set to the mean value of capital income share of circulation industry and its sub-sectors. The initial value of other parameters setting refers to the method of Klump et al. [45]. Using panel data of China’s circulation industry and sub-sector from 2004 to 2018, generalized nonlinear least squares estimation is carried out, and finally, the regression results in Table 1 are obtained. The table lists the parameter estimation result of the circulation industry and each sub-sector using the standardized supply-side system models.

**Table 1:** Parameter estimation results of circulation industry and sub-sector from 2004 to 2018 using the standardized supply-side system model.

Parameter	ξ	σ	α	γ_L_	γ_K_
Circulation industry	1.020*** (0.004)	0.300*** (0.015)	0.718*** (0.005)	0.083*** (0.004)	-0.028*** (0.002)
Transportation, storage and postal industry	0.993*** (0.005)	0.347*** (0.007)	0.855*** (0.003)	0.0213** (0.009)	-0.0106*** (0.009)
Wholesale and retail industry	0.980*** (0.006)	0.430*** (0.014)	0.637*** (0.010)	0.065*** (0.005)	-0.058*** (0.005)
Accommodation and Catering industry	0.978*** (0.006)	0.432*** (0.013)	0.731*** (0.007)	0.039*** (0.007)	-0.023*** (0.006)

Notes

*, **, *** indicate significance at the 10%, 5%, and 1% significance levels, respectively, and the standard errors of the estimated coefficients are in parentheses.

All estimated parameter results are significant at 1% level. The scale factor ξ of the circulation industry is 1.020, which is very close to 1. The substitution elasticity σ of labor and capital in circulation industry is 0.30, which is significantly less than 1, indicating that the relationship between capital and labor in circulation industry in China is complementary. The capital intensity α is 0.718, which is close to the sample average value of the capital gain share in circulation industry of 0.7. The growth parameter γL of labor efficiency is greater than 0, which is 0.083, implying that the growth rate of labor efficiency in circulation industry is positive. While the growth parameter of capital efficiency γK is less than 0, which is -0.028, indicating that the growth rate of capital efficiency in circulation industry is actually negative. The various parameters of circulation industry’s sub-sectors are all in line with reality. Among them, the transportation, storage and postal industries have the largest capital intensity at 0.855, followed by the accommodation and catering industries, and finally the wholesale and retail industries. Therefore, according to Formula (5), we can use the estimated values of substitution elasticity and capital intensity in Table 1, and combine the data of circulation industry output, labor and capital input to calculate biased technical progress index in China’s circulation industry and sub-sector from 2004 to 2018.

It can be derived from Table 2 that from 2004 to 2018, the sample average value of biased technical progress index of China’s circulation industry is 0.009, and its value is greater than 0. Although some provinces’ biased technical progress index is negative in several years, the overall mean value of the circulation industry is positive, which represents that the technical progress of China’s circulation industry is biased toward the capital. The biased technical progress index of the circulation industry’s sub-sector shows that the technical progress of the three sub-sectors is biased towards the capital. The wholesale and retail industry has the lowest degree of capital bias, and transportation, storage and postal industry has the highest. This is because compared with the other two sub-sectors, transportation, storage and postal industry has relatively large capital investment and a relatively large share of capital; When technical progress is biased towards capital, the degree of this bias will be even higher.

**Table 2:** Descriptive statistics of the biased technical progress index of circulation industry and sub-sector.

Variable	N	Mean	Std. Dev.	Min	Max
Circulation industry	420	0.009	0.698	-3.949	6.999
Transportation, storage and postal industry	420	0.101	0.503	-1.042	5.727
Wholesale and retail industry	420	0.0002	0.448	-0.960	3.993
Accommodation and Catering industry	420	0.041	0.623	-0.703	7.578

Notes: This paper adopts the data of 30 provincial administrative units in China from 2004 to 2018 as research sample, with a total of 450 sample data, but the sample size of the biased technical progress index is 420. Since the biased technical progress index (Eq 5) depends on substitution elasticity and change rate of capital-labor marginal output ratio, the calculated biased technical progress index will reduce the sample data of one period.

## 5. Empirical analysis of the influence of biased technical progress on employment in China’s circulation industry

### 5.1 Model setting

For the purpose of exploring the effect of biased technical progress of circulation industry on employment, this paper takes the employment scale of the circulation industry as the explained variable, the biased technical progress in circulation industry as the explanatory variable, the economic development level, capital level, and labor income level of circulation industry in all provinces, urbanization level, foreign trade level, industrial structure, foreign direct investment level and education level in all provinces as the control variables. The Hausman test is passed in advance, and the p-value is 0.000, and the test result is significant. Therefore, the null hypothesis of random effects model is rejected, and we select the fixed effects model. Set up the following model:

$$L_{i,t}=\theta_{0}+\theta_{1}D_{i,t}+\theta_{2}X_{i,t}+\eta_{i}+\mu_{t}+\varepsilon_{i,t}$$ (14)

where L_i,t_ is the employment scale of the circulation industry, D_i,t_ is the biased technical progress index, and i represents the province, t represents the time, X_i,t_ is the control variable, including the economic development level, capital level, and labor income level of circulation industry in each province, urbanization level, foreign trade level, industrial structure, foreign direct investment level and education level in each province. η_i_, μ_t_ and ε_i,t_ represent regional fixed effects, time fixed effects, and random disturbance terms, respectively.

### 5.2 Variable description

#### Explained variable

The employment scale (L_i,t_) is expressed by the number of employees in circulation industry at the end of the year in each province.

#### Core explanatory variables

The biased technical progress index (*D*_i,t_) of circulation industry uses the data obtained in the fourth part of this paper.

#### Control variable

The control variables (X_i,t_) include the economic scale, capital level, labor income level, urbanization level, foreign trade level, industrial structure, foreign direct investment level, and education level in each provinces’ circulation industry. The detailed measurement method is as follows: The level of economic development (Y_i,t_) is expressed by the value-added of circulation industry and sub-sectors. The level of capital (K_i,t_) is expressed by the capital stock of each province’s circulation industry and sub-sector. The labor income level (w_i,t_) is expressed by the average wage of each province’s circulation industry and sub-sectors. If labor costs are too high, enterprises will choose lower-cost capital to replace labor, leading to a decline in employment in the circulation industry. The urbanization level (City_i,t_) is expressed by the urbanization rate of permanent population in each province. The urbanization rate uses the ratio of the permanent urban population at the end of the year in each province. Because the rapid development of urbanization can absorb the surplus labor transferred from traditional agriculture and the second industry, bringing about an increase in employment ratio of second industry, and circulation industry has great advantages in absorbing this non-knowledge intensive labor force. Since the state revised the statistical standards in 2015, the statistical caliber was changed from non-agricultural population and agricultural population to urban population and rural population. However, since no specific changed statistical standards are available, this paper uses urban population instead of non-agricultural population. The level of foreign trade (Export_i,t_ and Import_i,t_) is expressed by the import and export in each province. Because import and export have different effects on the circulation industry’s employment, the level of foreign trade is divided into two indicators: import and export. Simultaneously, the average annual exchange rate of RMB to 100 US dollars is used to convert the import and export volume into an amount in RMB. The industrial structure (Structure_i,t_) uses the proportion of the value-added of third industry in each province to GDP. The industrial structure, especially the proportion of third industry, has become an important factor for employment in circulation industry. The level of foreign direct investment (FDI_i,t_) is expressed by the amount of foreign direct investment in each province. We calculate the amount of foreign direct investment at a constant price in 2004 by using the GDP deflator to measure the investment attraction of each province in China. The influence of foreign direct investment on employment in China’s circulation industry has two effects, including “creation effect” [46] and “destructive effect”. The level of education (Edu_i,t_), which is the most important factor in determining employment, is expressed by years of education per capita of 6 years old or older in each region. Based on the calculation method and actual situation of the existing literature, the number of years of education in primary school, junior school, high school, and junior college and above before 2015 is recorded as 6, 9, 12, and 16 years, respectively. And the education level is: Edu = 6*Primary school+9*Junior school+12*High school+16*Junior college and above, among which primary school, junior school, high school, junior college and above indicate their proportion of the population over 6 years old, respectively. After 2015, the years of education in primary school, junior school, general high school, secondary vocational school, junior college, university, and postgraduate are recorded as 6 years, 9 years, 12 years, 12 years, 15 years, 16 years, and 19 years, respectively. The level is: Edu = 6*Primary school+9*Junior school+12*General high school+12*Secondary vocational school+15* Junior college+16* university +19* Postgraduate, among which primary school, junior school, general high school, secondary vocational school, junior college, university and postgraduates respectively indicate their proportion of the population over 6 years old. The number of years of education without going to school is 0 years, so the number of this part of the population is not included in the average years of education per capita.

### 5.3 Data source

This paper adopts 30 provincial panel data from 2004 to 2018. The data stems from the 2005–2019 China Statistical Yearbook, China Population and Employment Statistical Yearbook, China Labor Statistics Yearbook, and 30 provincial administrative units’ statistical yearbook. Data for some provinces are missing, so we adopt the available associated data to calculate and fill up; if still missing, we use interpolation or regression to fill up.

### 5.4 Cross-sectional dependence tests

In order to avoid estimation bias and size distortion caused by possible cross-sectional dependence in the panel model [47], this paper refers to the study of Li et al. [48], and adopts Pesaran scaled LM test and Frees test to investigate whether there is cross-sectional dependence in the panel data.

Table 3 shows the results of two cross-sectional dependence tests, namely the Pesaran scaled LM test and the Frees test. As shown in Table 3, the statistical data of both tests significantly reject the null hypothesis, providing solid evidence for cross-sectional dependence among Chinese provinces. Therefore, when using this panel sample for further analysis, we use estimation techniques that allow cross-sectional dependence [49].

**Table 3:** Results of the cross-sectional dependence tests.

Test	Statistics
Pesaran scaled LM test	2.366**
Frees test	5.718***

Notes

*, **, *** indicate significance at the 10%, 5%, and 1% significance levels, respectively.

### 5.5 Benchmark regression analysis

Table 4 shows the regression results of the influence of biased technical progress index on the employment scale of circulation industry. Models (1)-(3) are the regression results of fixed effects models. Among them, model (1) is the influence of individual regression biased technical progress on the employment scale of circulation industry, and model (2) adds the control variables of circulation industry based on model (1). Model (3) adds the remaining control variables based on model (2).

**Table 4:** Regression results of the influence of biased technical progress on the employment scale of circulation industry.

	(1)	(2)	(3)
D	-0.042*** (-3.361)	-0.049*** (-4.020)	-0.044** (-2.45)
The level of economic development		0.180** (2.581)	0.083 (1.191)
Capital level		-0.192*** (-2.968)	-0.110 (-1.576)
Labor income level		-0.347*** (-3.193)	-0.341*** (-3.182)
Urbanization level			0.005 (0.990)
Export level			0.100*** (3.429)
Import level			-0.070*** (-2.927)
Industrial structure			0.009*** (2.773)
Level of foreign direct investment			0.011 (0.732)
Education level			0.073 (1.640)
Time FE	YES	YES	YES
Regional FE	YES	YES	YES
Hausman test value	-1280.40 [0.000]	101.19 [0.000]	104.64 [0.000]
Constant term	5.495*** (31.128)	5.868*** (5.114)	6.426*** (5.204)
N	420	420	420
R^2^	0.872	0.881	0.893

Notes

*, **, *** indicate significance at the 10%, 5%, and 1% significance levels, respectively. The t-values of the estimated coefficient is in parentheses, and the P-value is in square brackets.

From the regression results in Table 4, it can be derived that the relationship between biased technical progress index and employment scale of circulation industry is negatively correlated, and it is significant at a significance level of 5%. It implies that capital-biased technical progress has a “destructive effect”. Therefore, with the increase of biased technical progress index, the employment scale of circulation industry will shrink. It can be deduced from the regression coefficient of biased technical progress in model (3) that for every 1% increase in the biased technical progress index, the employment scale of circulation industry will decrease by 0.044%. The increase in the output of circulation industry is not greater than the increase in labor efficiency, which results in a decrease in employment in the circulation industry.

Explanation of the control variables: In model (2), the coefficient of value-added in the circulation industry is significantly positive at 5% significance level. It implies that the increase in value-added in circulation industry has a promoting effect on the scale of employment. Specifically, for every 1% increase in the circulation industry’s value-added, the circulation industry’s employment scale will correspondingly increase by 0.180%. The capital stock of the circulation industry has a negative correlation with the employment scale at a significant level of 1%. That is, for every 1% increase in the circulation industry’s capital stock, the circulation industry’s employment scale will shrink by 0.192%. Because the increase in capital stock accelerates capital deepening, the rate of capital growth is inconsistent with the rate of labor growth, and capital growth is faster than labor growth, which leads to a relative decline in labor [50]. The coefficient of labor wages is negative at 1% significance level. That is, as the wages of labor in circulation industry increase, enterprises will use less labor due to cost considerations, which will result in a decrease in employment.

In model (3), after adding all control variables, the research results imply that the relationship between exports and employment in the circulation industry is positively correlated. That is, for every 1% increase in the export value, the employment scale in circulation industry will increase by 0.1%. The export trade development will drive the increase of employment in circulation industry to a certain extent. Because when the domestic exports increase, a large amount of workforce is needed to transport products or services, so more employment opportunities in the circulation industry will be created. Imports are negative at a significance level of 1%. That is, imports and employment in the circulation industry are negatively correlated. Specifically, for every 1% increase in imports, the employment scale in the circulation industry will shrink by 0.07%. The increase of imports will crowd out the domestic market, intensify competition among domestic producers and reduce the domestic market share of enterprises. Domestic production enterprises will correspondingly reduce supply, further affecting circulation industry’s development and thus circulation industry’s employment scale.

The optimization of industrial structure has a significant positive influence on circulation industry’s employment. That is, as the proportion of third industry gradually increases, a large amount of labor is gradually transferred from the first and second industries to third industry. At the same time, employment in circulation industry accounts for nearly 60% of tertiary industry, so a larger part of the labor force will be transferred to circulation industry, promoting the expansion of employment in circulation industry.

### 5.6 Robustness analysis

#### Re-inspection of tailing treatment

Since the regression result of the model may be affected by the extreme values of each variable, on the premise of sufficient sample data, we carried out 1% tail reduction for all variables by referring to the study of Baker et al. [51], that is, extreme values processing was carried out at 1% and 99% quantiles. For numbers less than 1%, we assign 1% value, and for numbers greater than 99%, we assign 99% value, and then use new samples to conduct regression analysis again. The regression results are shown in Table 5, columns (1). The regression results show that capital-biased technical progress still has a significant negative influence on the employment scale of circulation industry. From this perspective, the benchmark regression result is robust.

**Table 5:** Robustness test and endogenous test results.

variable	Tailing treatment(1)	Replacement of the explanatoy variable’s measurement index(2)	Re-selecting the sample interval(3)		GMM (4)	Reverse causality(5)	Missing variables(6)
			2004–2011	2012–2018			
D	-0.031*** (-4.436)	-0.008*** (-3.404)	-0.058*** (-4.496)	-0.024*** (-3.285)	-0.087*** (-2.58)	-0.020*** (-3.483)	-0.018*** (-3.206)
Constant	0.910 (1.571)	0.621** (2.482)	3.917*** (3.007)	3.766*** (2.611)	-1.017 (-1.23)	0.048*** (2.664)	1.152* (1.863)
Control variable	YES	YES	YES	YES	YES	YES	YES
Anderson canon. corr. LM Statistics						57.276	
Cragg-DonaldWald F Statistics						22.017	
AR(1)					-2.68*** (0.007)		
AR(2)					-0.53*** (0.597)		
Hansen test					19.98 (0.334)		
Time FE	YES	YES	YES	YES	YES	YES	YES
Regional FE	YES	YES	YES	YES	YES	YES	YES
N	420	420	210	210	420	360	420
R^2^	0.709	0.772	0.832	0.493		0.121	0.699

Notes

*, **, and *** indicate significance at the 10%, 5%, and 1% significance levels, respectively. The t-values of the estimated coefficients are in parentheses

#### Re-inspection of the replacement of the explanatory variable’s measurement index

Different selected indicators of the explained variables may also lead to different results in the research conclusions, so the sensitivity of the indicators needs to be analyzed. This paper replaces the indicators that measure the employment scale of circulation industry with the proportion of employment in circulation industry to the number of employees in China and re-validates the model. The regression results are shown in Table 5, columns (2). The regression results show that the impact of the biased technical progress index on the scale of the circulation industry is still significantly negative, representing that capital-biased technical progress has a restraining effect on the employment growth of circulation industry. It also suggests that the benchmark regression result is robust.

#### Re-inspection of the re-selecting sample interval

Selecting data from different sample intervals may also produce different results. Since part of the data from 2004 to 2008 were calculated based on the comprehensive proportion, it may also bring about errors in the measurement results. Therefore, we redivide the time area into 2004–2011 and 2012–2018, and carry out the regression of the model again. The specific regression results are shown in Table 5, columns (3). The regression results show that the regression coefficient from 2004 to 2011 is -0.058, and the regression coefficient from 2012 to 2018 is -0.024, both of which are significant at 1% level. Therefore, the biased technological progress index has a significant negative impact on the employment scale of the circulation industry, and its impact on the employment structure of the circulation industry is not very different from the result of benchmark regression, which confirms that the benchmark regression results in this paper are robust.

#### Re-inspection of the replacement model

Considering that the variables selected in this paper have a long continuity at the time level, the past employment scale of the circulation industry may have an impact on the future employment scale, thus this paper adopts the dynamic panel model for regression. In this paper, the employment scale index with a lag of one period is added to the explanatory variables, and the two-step method of system GMM is used to estimate it. The model can effectively control the possible dynamic effects and avoid biased estimation, and the results obtained are relatively robust. Set up the following model:

$$L_{i,t}=\theta_{0}+\theta_{1}lnD_{i,t}+\theta_{2}lnD_{i,t-1}+\theta_{3}X_{it}+\mu_{i}+\lambda_{t}+\varepsilon_{it}$$ (15)

where lnD_i,t−1_ is the employment scale index with a lag of one period.

The prerequisite of the system GMM is that there is no second-order or higher autocorrelation in the residual terms of the difference equation, and the instrumental variables are exogenous, so the AR(1), AR(2) and Hansen test are carried out in this paper. Table 5 shows the regression results after replacing model, which can be obtained by the P-values of AR(1) and AR(2). The regression results all pass the autocorrelation test of the disturbance terms significantly. The residual sequence of the difference equation has first-order autocorrelation, but does not have second-order autocorrelation. At the same time, the P-value of Hansen test is greater than 0.1, and the hypothesis that all the instrumental variables are exogenous is accepted. The dynamic panel model is set reasonably, and the estimation results of system GMM are valid.

### 5.7 Endogenous analysis

#### Reverse causality

The problem of reverse causality involved in this paper’s research stems from two aspects. First, there is a reverse causality between explained variable and explanatory variable, that is, while capital-biased technical progress has a inhibitory effect on the employment of circulation industry, the employment of circulation industry also has an impact on the capital-biased technical progress. Second, there is a reverse causal relationship between explained variable and control variable. For example, the employment of circulation industry is affected by the economic development level and capital level of circulation industry, while the employment in circulation industry affects the economic development level and capital level of circulation industry.

For the purpose of solving the above endogenous problems, we adopt instrumental variables method. We take the lag period of the biased technical progress index, the lag period of the added value of circulation industry and the lag period of capital stock as the instrumental variables of current values. This is a very common method to select instrumental variables in panel data models. The above three lag variables (instrumental variables) have a strong correlation with the current values, while the current circulation employment has no influence on the above three lag variables, which conforms to the selection principle of instrumental variables. According to the estimation results in Table 5, the symbol and significance of estimation coefficient after the use of instrumental variables are consistent with the baseline regression, while the absolute value of estimation coefficient is smaller than the baseline regression, and the Anderson canon. corr. LM statistic and the Stock-Yogo weak IV test critical value show that the tool variable passes the “weak tool variable” and “unrecognized” tests, so it is proved that the instrumental variables selected in this paper is effective. Therefore, the results of the benchmark regression still hold after considering the endogeneity issue, so it is reliable to conclude that the impact of biased technological progress on employment in circulation industry is significantly negative.

#### Missing variables

In order to solve the problem of missing variables, this paper refers to the practice of existing literature, adds more control variables to the original model, and observes the coefficient of biased technical progress. Among the factors affecting employment in circulation industry, in addition to the development of circulation industry itself and China’s economic development capabilities, the technical innovation capabilities, the infrastructure construction, and the degree of marketization also have a certain effect on employment in circulation industry. Therefore, based on the benchmark regression, this paper controls the indicators of technical innovation capability (the logarithm of the number of domestic patent applications granted), infrastructure construction (the logarithm of the number of road miles owned by every 10,000 people), and the degree of marketization (the proportion of investment in the non-state-owned units = (the fixed asset investment of the whole society-the fixed asset investment of the state-owned economy)/the fixed asset investment of the whole society). The empirical results are shown in Table 5, columns (5), the benchmark regression results are still robust.

## 6. Further analysis

Next, from the perspective of heterogeneity, this paper explores the influence of biased technical progress on the employment scale of circulation industry’s different ownerships, different levels of education, urban and rural areas, different sub-sectors, and different regions.

### 6.1 Different ownerships

Different ownerships are divided into state-owned units and non-state-owned units. The explained variables are the proportion of employees in state-owned units and non-state-owned units in circulation industry to the number of employees in third industry. Panel A in Table 6 describes the regression results of ownership structure. It can be seen that the coefficient of biased technical progress index has a negative impact on the employment scale of circulation industry of non-state-owned units at a significance level of 1%. Specifically, for every 1% increase in biased technical progress index, the employment share of non-state-owned units in circulation industry decreases by 0.019%. The possible reasons for the research results are that state-owned enterprises are protected by policies and have sufficient guarantee capacity to obtain funds from financial institutions. The labor costs of state-owned units are relatively high and are strictly protected by law. Even if there is capital-biased technical progress, the employment of state-owned units will not be greatly affected. Non-state-owned units have certain thresholds when they obtain funds, so the cost of capital factors of such units is very high, and the market spontaneously adjusts the labor supply and demand of non-state-owned units. Simultaneously, the labor cost of such units is relatively low. Since the substitution elasticity of capital and labor in the circulation industry is less than 1, the price effect plays a greater role at this time, and technical progress is manifested as a greater preference for scarce elements. That is, the technical progress of non-state-owned units can further the growth of marginal output of capital to a greater extent, resulting in a decrease in employment of non-state-owned enterprises in circulation industry.

**Table 6:** Heterogeneity analysis regression results.

variable	Panel A				Panel B				Panel C			
	State- owned unit		Non-state-owned unit		Low level of education		High level of education		City		Rural	
D	0.000 (0.321)		-0.019*** (-3.427)		-0.015*** (-3.313)		-0.004** (-2.534)		-0.034*** (-3.639)		-0.003 (-0.922)	
Constant	0.047 (1.208)		1.299** (2.075)		0.781 (1.530)		0.598*** (3.469)		3.326*** (3.135)		-0.621 (-1.567)	
Control variable	YES		YES		YES		YES		YES		YES	
Time FE	YES		YES		YES		YES		YES		YES	
Regional FE	YES		YES		YES		YES		YES		YES	
N	420		420		420		420		420		420	
R^2^	0.787		0.714		0.593		0.810		0.697		0.319	
variable	Panel D					Panel E						
	Transportation, storage & postal industry	Wholesale and retail industry		Accommodation and Catering Industry		East		Central		West		North- east
D	-0.004*** (-4.884)	-0.039*** (-5.581)		-0.007*** (-5.472)		-0.065*** (-2.656)		-0.010 (-0.979)		-0.037*** (-3.819)		0.001 (0.221)
Constant	0.542*** (8.801)	0.971** (2.267)		-0.199* (-1.859)		3.225** (2.026)		3.215* (1.831)		-0.028 (-0.034)		-1.235 (-0.537)
Control variable	YES	YES		YES		YES		YES		YES		YES
Time FE	YES	YES		YES		YES		YES		YES		YES
Regional FE	YES	YES		YES		YES		YES		YES		YES
N	420	420		420		140		84		154		42
R^2^	0.377	0.658		0.697		0.726		0.853		0.800		0.967

Notes: (1) *, **, and *** indicate significance at the 10%, 5%, and 1% significance levels, respectively. The t-values of the estimated coefficients are in parentheses; (2) In the ownership structure, the coefficient of the biased technical progress index of state-owned units is-0.000, because the data in this paper only retains three decimal places, and its actual value is 0.000112.

### 6.2 Different levels of education

Different levels of education are divided into low levels of education and high levels of education. Low levels of education include illiteracy, elementary, junior high and high school levels education. The high level of education includes college, undergraduate and postgraduate education. The explained variables are the proportion of the number of employees with low education and high education levels in circulation industry to the number of employments in third industry. Panel B in Table 6 describes the regression results of educational structure. According to the standards of the National Bureau of Statistics before 2015, it is divided into seven categories: illiteracy, primary school, junior school, high school, junior college, university, and postgraduate. Labor Force Survey since 2015 to start using the new classification of educational attainment: no schooling, primary school, junior school, high school, secondary vocational school, advanced vocational school, junior college, university and postgraduate nine categories. This paper equates no schooling after 2015 to illiteracy before 2015, divides the total of high school and secondary vocational school into high school before 2015, and divides the total of advanced vocational school and junior colleges into junior college before 2015. It can be derived from the table that biased technical progress has a negative influence on employment at high and low levels of education and has a greater influence on employment at low levels of education. The specific result is that the coefficient of the influence of capital-biased technical progress on employment of low-educational circulation industry is -0.015. For every 1% increase in biased technical progress index, the proportion of employment in circulation industry with low education level will decrease by 0.015%. The employment influence of a high level of education in circulation industry is -0.004. That is, for every 1% increase in the biased technical progress index, the proportion of employment with a high level of education in circulation industry will decrease by 0.004%. The possible reason is that due to the law of diminishing margins, the labor efficiency growth parameter of employees with low education levels is higher, while the labor efficiency growth parameter of employees with high education levels is lower. Therefore, when the output of the circulation industry does not increase sufficiently, it will “destroy” the employment of people with low education levels to a greater extent. The another possible reason for the greater damage of capital-biased technical progress to the employment at low levels of education in circulation industry is that the skills of employees with high education is higher than employees with low education. The deepening of capital accumulation will increase the demand of high-skilled labor force of enterprises through the combination of advanced machinery equipment and high-skilled labor force, as well as the reform of management and organization of enterprises. It is manifested as skill-biased technical progress that offsets the destructive effect of capital-biased technical progress on the employment of people with low education levels.

### 6.3 Urban and rural areas

Urban and rural areas refer to towns and villages, respectively. Urban employment in the circulation industry includes urban state-owned enterprises, collective enterprises, private enterprises, other enterprises, and individuals. Rural employment in the circulation industry includes rural private enterprises and individual employees. Among them, the number of rural employees in circulation industry = the number of employees in circulation industry at the year’s end by private enterprises and individuals-the number of urban private enterprises and individual employees in circulation industry. The explained variables are the proportion of urban and rural employment in circulation industry to the number of employments in third industry. Panel C in Table 6 describes the regression results of urban-rural structure. As evidenced by the table that the increase of biased technical progress index has significantly reduced the number of urban employments in circulation industry, and the influence on rural employment is not significant. From the results of urban employment, it can be seen that the elasticity of urban employment in circulation industry to the biased technical progress index is -0.034, which is significant at 1% significance level. The possible reason is: On the one hand, the increase in the output of urban circulation industry is relatively small, which makes its growth rate lower than the increase in labor efficiency, thus leading to a decrease in employment in urban circulation industry. The output of rural circulation industry has increased significantly, and its increase is similar to the increase in labor efficiency, so the employment of rural circulation industry will not decrease significantly.

On the other hand, China’s actual economic situation is that the differences in the accumulation of urban and rural factors are extremely obvious. The capital factors in rural areas are relatively scarce, and the capital-biased technical progress mainly occurs and/or is adopted in urban areas. While rural areas are restricted by infrastructure, factor endowments and the level of economic development, the technical progress occurs and is adopted slowly, preferring to use traditional technologies for a long time. Therefore, the rural areas will not be susceptible to the capital-biased technical progress and the demand for labor force in rural circulation industry is stable.

### 6.4 Different sub-sectors

This paper divides the circulation industry into three sub-sectors: transportation, storage and postal, wholesale and retail, and accommodation and catering. According to the data of the sub-sectors, the corresponding biased technical progress index is calculated, and re-regress the sample by sub-sectors. The explained variables are the proportions of the employment in the three sub-sectors to the employment in the third industry. Panel D in Table 6 describes the empirical results of industry structure. The findings indicate that three sub-sectors are negatively impacted by biased technical progress index. That is, the increase of biased technical progress index will cause the decrease of employment in each sub-sector, and it is at a significant level of 1% significant. However, the degree of influence on different industries is not the same. It is manifested that capital-biased technical progress has the greatest influence on wholesale and retail industry. The coefficient of employment to biased technical progress is -0.039. Followed by the accommodation and catering industry, the coefficient is -0.007. The least influence on transportation, storage and postal industry is -0.004. The reasons may be: When the increase in output is not significant enough, the labor efficiency of wholesale and retail industry has the fastest growth (γL = 0.065), while the labor efficiency of transportation, storage and postal industries has the slowest growth (γL = 0.0213). Therefore, the “destructive effect” on employment in wholesale and retail industry is the largest, while the “destructive effect” on employment in transportation, storage and postal industries is the smallest.

### 6.5 Different regions

Different regions are classified into the east region, middle region, west region and the northeast region according to the geographical regions divided by the country. According to the statistical system and classification standards promulgated by the National Bureau of Statistics, China’s economic belts are divided into four economic belts: the eastern region, the central region, the western region, and the northeast region. The specific division is: the eastern region covers 10 provinces (cities) including Beijing, Tianjin, Hebei, Shanghai, Jiangsu, Zhejiang, Fujian, Shandong, Guangdong and Hainan; the central region covers 6 provinces (cities) including Shanxi, Anhui, Jiangxi, Henan, Hubei and Hunan; The western region covers 11 provinces (cities) including Inner Mongolia, Guangxi, Chongqing, Sichuan, Guizhou, Yunnan, Shaanxi, Gansu, Qinghai, Tibet, Ningxia and Xinjiang; the northeastern region includes Liaoning, Jilin and Heilongjiang. Due to the serious lack of data in Tibet, Tibet is not included in the statistics of the western region. The explained variables are the proportion of employment in circulation industry in eastern, central and western regions to the employment in tertiary industry in northeastern region. Panel E in Table 6 describes the regression results of regional structure. The estimated results indicate that under the circumstance that the control variables remain unchanged, capital-biased technical progress negatively influences the employment of circulation industry in eastern and western regions. However, the degree of influence is different, the influence on eastern region is greater, and the influence on western region is relatively small. The influence on employment in circulation industry in central and northeastern regions is not significant. The circulation industry employment impact of biased technical progress on eastern region’s is -0.065. That is, for every 1% increase in the biased technical progress index, the proportion of employment in the eastern region will shrink by 0.065%. The influence of biased technical progress on employment in circulation industry in western region is -0.037. That is, for every 1% increase in the biased technical progress index, the proportion of employment in the western region will shrink by 0.037%. The possible reasons are: the low level of economic development, backward production technology, and low education level of the labor force have affected the development of technical progress in western region. Historically, the region has developed more labor-intensive industries with lower technical levels, and the circulation industry has more room for development in the region. This has promoted the increase in employment demand in circulation industry in the region. When capital-biased technical progress occurs, the western region is restricted by objective conditions, resulting in insufficient capital deepening and a lower degree of capital crowding out of labor. In the eastern region, due to regional advantages and policy advantages, capital deepening can be formed quickly when technical progress is biased towards the capital. Because technical progress is biased towards the capital, the deeper the degree of capital deepening, the greater the degree of capital crowding out of labor.

## 7. Conclusions and policy recommendations

This paper uses the China’s industry data in 2004–2018 to measure the biased technical progress in circulation industry of China and studies the influence of biased technical progress index on the scale of employment in circulation industry. The research conclusions are as follows:

First, from 2004 to 2018, the technical progress of China’s circulation industry and sub-sectors was biased toward capital, and the relationship between capital and labor in circulation industry and sub-sectors was complementary. Second, from 2004 to 2018, the relationship between capital-biased technical progress and employment scale of circulation industry in China is negatively correlated, and it is significant at 5% significance level. Both the robustness analysis and endogenous analysis demonstrate that the benchmark regression results are robust. Third, the analysis of heterogeneity in this paper shows that: At the ownership level, capital-biased technical progress has a significant negative influence on the employment of non-state-owned units, but has no significant influence on state-owned units. At the educational level, the employment of high and low-educated employees in the circulation sector is more adversely impacted by capital-biased technical progress. However, it has a greater negative influence on employees with low education. At the urban-rural level, the bias technology progress index has a significant negative influence on urban employment in the circulation industry but has no significant influence on rural employment. At the sub-sectors industry level, the influence of capital-biased technical progress on three sub-sectors is significantly negative, but there is a big difference in the influence coefficient. The influence on wholesale and retail industry is greater, and the influence on transportation, storage and postal industry is less. At the regional level, capital-biased technology progress has a negative influence on employment in eastern and western regions and is significant at 1% significance level, but it has no significant influence on employment in central and northeast areas.

According to the above research conclusions, in order to effectively utilize and advance the technical progress of circulation industry, improve the quality of economic growth, and achieve the goal of full employment, this paper puts forward the following targeted policy suggestions:

First, optimize the structure of circulation industry. The capital-biased technology progress has little negative impact on transportation, storage and post industry, accommodation and catering industry. Therefore, to expand the employment of the flow industry, we should focus on the development of these two sub-sectors of circulation industry. At the same time, the wholesale and retail industry should develop labor-biased technical progress, promote the absorption of employment, and expand labor demand. Second, we will deepen market-oriented reform of interest rates. When the currency market is a completely market-oriented currency market, capital will form a reasonable price under an effective pricing mechanism, and circulation enterprises will choose production technologies that conform to factor endowments. The government’s inappropriate intervention in currency market and regulation on the interest rate will make the interest rate signal distorted and the transmission channel of interest rate blocked, then the capital lacks an effective pricing mechanism, resulting in the low relative price of capital factor and stimulating enterprises to use considerable labor factors, which will lead to the excessive use of capital-biased technical progress. Therefore, there is a need to promote the market-oriented reform of interest rates and constantly build and improve the market-oriented interest rate system, encourage circulation enterprises to choose production technologies that conform to factor endowments, promote the input of various factors of enterprises to reach the optimal level, improve production efficiency, boost economic development, and avoid the negative impact of the blind selection of capital-biased production technology on the labor market. Third, make reasonable use of regional advantages and fully absorb employment. To strengthen the ability of the circulation industry to absorb employment, we should leverage the advantages of eastern and central regions fully to promote the expansion of employment scale and improve the level of social employment. Fourth, improve the education level and promote the skill enhancement of the labor force. On the one hand, by associating school knowledge education with industrial skill training effectively, the cultivation of high-skilled labor force can be enhanced to meet the demand of high-skilled labor force causing by the skilled-biased technical progress in circulation industry, and the opportunities for labor force to enter the circulation can be increased. On the other hand, the skill-biased characteristics of the technical progress in circulation industry can improve the overall labor productivity of circulation enterprises through the increase of the proportion of high-skilled labor force, thus promoting the expansion of enterprise scale and further increasing employment opportunities. Fifth, improve the credit system. To establish a relatively fair credit market, we should no longer discriminate between different enterprises, make non-state-owned circulation enterprises and state-owned circulation enterprises, rural circulation enterprises and urban circulation enterprises receive the same financial support, ensure the prosperity of non-state-owned circulation enterprises and rural circulation markets, research and develop or adopt labor-biased technical progress in accordance with factor endowments, further alleviate employment pressure and increase employment positions.

## Supporting information

S1 Appendix (XLSX)
